# Psychosis as a symptom of Vitamin B12 deficiency. Report of one case

**DOI:** 10.1192/j.eurpsy.2021.1126

**Published:** 2021-08-13

**Authors:** A. Calle-Gonzalez, D. Batet-Sanchez, A. Hernández Mata, A. Sotillos Gómez, R. Pinilla Zulueta

**Affiliations:** Psychiatry, Hospital Universitario de Getafe, Getafe, Spain

**Keywords:** Vitamin b12, Cyanocobalamin, dementia, psychosis

## Abstract

**Introduction:**

Vitamin B12 deficiency may cause neurological and psychiatric symptoms, especially among elderly patients. Two clinical cases are presented of patients admitted to an Acute Inpatient Psychiatry Unit due to psychotic symptoms, being reported a B12 deficiency.

**Objectives:**

Review clinical information about vitamin B12 deficiency as a factor involved in the development of psychiatric disorders, specifically psychotic symptoms, pointing out the peculiarities regarding clinical presentation, diagnosis, prognosis, and treatment management.

**Methods:**

Search in the medical database PUBMED, MEDSCAPE and UPTODATE.

**Results:**

Vitamin B12 deficiency is associated with hematological, neuropsychiatric, and digestive disorders, is estimated that around 5-40% of the elderly population may present it. Neuropsychiatric syndromes may be the first, and sometimes sole, manifestation, related to a different etiological mechanism. Vitamine B12 deficiency implies enzymatic defects that cause an accumulation of methylmalonic acid and homocysteine, which is proportionally related to the severity of the neuropsychiatric symptoms. The range of clinical features includes psychotic and affective episodes, behavioral disorders, cognitive impairment, along with other neurological manifestations such as polyneuropathy and encephalopathy. The diagnosis delay is crucially important, as early detection could lead to reverse the neuropsychiatric symptoms and some of the neuroradiological alterations. Parenteral and oral vitamin B12 supplementation should be initiated, monitoring levels in plasma, together with psychiatric drugs until the symptoms are controlled.

**Conclusions:**

Vitamin B12 deficiency is a factor that may be involved in the etiopathogenesis of psychiatric disorders. Thus, screening must be considered among the vulnerable population when presenting neuropsychiatric disorders as early diagnosis and treatment are key to clinical prognosis.

